# The Genome Sequence of an Oxytetracycline-Resistant Isolate of the Fish Pathogen *Piscirickettsia salmonis* Harbors a Multidrug Resistance Plasmid

**DOI:** 10.1128/genomeA.01571-16

**Published:** 2017-02-02

**Authors:** Harry Bohle, Patricio Henríquez, Horst Grothusen, Esteban Navas, Fernando Bustamante, Patricio Bustos, Marcos Mancilla

**Affiliations:** Laboratorio de Diagnóstico y Biotecnología, ADL Diagnostic Chile SpA, Puerto Montt, Chile

## Abstract

The amount of antibiotics needed to counteract frequent piscirickettsiosis outbreaks is a major concern for the Chilean salmon industry. Resistance to antibiotics may contribute to this issue. To understand the genetics underlying *Piscirickettsia salmonis*-resistant phenotypes, the genome of AY3800B, an oxytetracycline-resistant isolate bearing a multidrug resistance plasmid, is presented here.

## GENOME ANNOUNCEMENT

Salmon farming is one of the main economic activities of Chile. However, diverse epizootics have constantly been hampering its sustainability and development. Piscirickettsiosis, also known as salmonid rickettsial septicemia (SRS), deserves special attention, since it has shown remarkable resilience to being controlled. The disease has high incidence and mortality in seawater-reared salmonids (1). Due to persistent outbreaks and low efficacy of vaccines, the use of antibiotics has scaled up. Official data indicate that over 500 metric tons of antimicrobials have been applied to treat SRS in 2015. Hence, the disease impact does not only result from the mortality but also from the environmental challenge arising because of the amount of antibiotics employed.

The above-described situation probably influenced the wild-type population of the etiological agent, the Gram-negative intracellular facultative bacterium *Piscirickettsia salmonis*, and triggered the selection of resistant types (2). The resistance to oxytetracycline (OTC) displayed by some isolates is particularly alarming, because the MIC observed *in vitro* by far exceeds maximum plasma levels recorded *in vivo* (3). This difference predicts a failure of OTC treatments.

Isolate AY3800B, with an MIC to OTC of 256 µg/ml, was recovered from Atlantic salmon (*Salmo salar*) during an SRS outbreak in 2013, in southern Chile. Sequencing was performed at Macrogen, Inc. (Seoul, South Korea) using the Pacific Biosciences (PacBio) SMRT cell 8Pac version 3 and DNA polymerase binding kit P6 version 2 for library preparation. An additional Nextera library was prepared to be run on an Illumina HiSeq 2000 platform. A total of 57,096 PacBio mapped reads (15,382 bp average length) with an *N*_50_ of 23,273 bp from a total of 60,443 (107× coverage) were *de novo* assembled into a circular chromosome of 3,187,887 bp, and four plasmids of 60,086 bp (p1PS10), 33,556 bp (p2PS10), 45,767 bp (p3PS10), and 188,301 bp (p4PS10) using SMRT Analysis version 2.3.0.1 (PacBio). The sequences were checked for errors by mapping Illumina paired-end reads (116,881,806 reads, 101 bp in average length, 1,510× coverage) over the genome built with PacBio reads. The data were annotated with the NCBI Prokaryotic Genome Annotation Pipeline using the best-placed reference protein set as the annotation method implemented in GeneMarkS+ revision 3.0 software resulting in 3,539 genes, 3,274 coding sequences (CDSs), 190 pseudogenes, six complete sets of rRNA genes, one small noncoding RNA (ncRNA), and 56 tRNAs. The chromosome depicts a G+C content of 39.73% (Table 1).

**TABLE 1:** Features of *P. salmonis* AY3800B replicons

Replicon	Length (bp)	G+C content (%)	No. of genes	No. of CDSs	No. of rRNAs	No. of tRNAs	Accession no.
Chromosome	3,187,887	39.73	3,149	3,031	18	56	CP013816
p1PS10	60,086	37.73	67	66	0	0	CP013817
p2PS10	33,556	40.62	43	41	0	0	CP013818
p3PS10	45,767	55.04	52	52	0	0	CP013819
p4PS10	188,301	39.14	228	222	0	0	CP013820

*P. salmonis* AY3800B belongs to the LF-89-like genogroup (4). Consistently, the same number of rRNA and tRNA genes, as well as iron scavengers and secretion systems previously observed in the LF-89 type strain, were identified (5). Interestingly, plasmid p3PS10 was found to be unique to the AY3800B isolate. p3PS10 is related to the multidrug resistance plasmid pEIB202 carried by *Edwarsiella tarda* (6). Additionally, p3PS10 contains a *tet* resistance determinant similar to the one found in the plasmid pRAS1, which conferred resistance to tetracyclines to a series of *Aeromonas salmonicida* isolates recovered in Norway (7). Further research is required to prove this hypothesis.

This sequence will allow for an assessment of the epidemiology and management of this resistant phenotype.

### Accession number(s).

Chromosome and plasmid sequences of *P. salmonis* AY3800B have been deposited at DDBJ/EMBL/GenBank under the accession numbers listed in Table 1. This paper describes the first version.
